# Comparative analysis of high-intensity resistance training and blood flow restriction training on enhancing upper limb muscle strength and mass

**DOI:** 10.3389/fphys.2025.1568616

**Published:** 2025-07-07

**Authors:** Jianli Zhang, Hualong Chang, Huijuan Cui, Biao Chen

**Affiliations:** ^1^ College of Physical Education and Health Sciences, Zhejiang Normal University, Jinhua, China; ^2^ Renji College, Wenzhou Medical University, Wenzhou, China; ^3^ Students' Affairs Office, Wenzhou Polytechnic, Wenzhou, China

**Keywords:** blood flow restriction, high-intensity resistance, cuff pressure, muscle strength, muscle mass

## Abstract

**Purpose:**

To investigate the effects of blood flow restriction training with fixed pressure combined with low-intensity resistance training (BFRT-F) and progressive pressure combined with low-intensity resistance training (BFRT-P) in enhancing upper limb muscle strength and mass, and to compare their effects with high-intensity resistance training (HIRT).

**Methods:**

A stratified randomized controlled trial was conducted, where 34 participants were randomly assigned to the HIRT, BFRT-F, and BFRT-P groups. The 8-week intervention included thrice-weekly training sessions.

**Results:**

1) All groups showed significant 1RM increases, with HIRT and BFRT-P superior BFRT-F. 2) HIRT significantly improved isokinetic muscle strength indicators, including peak torque of shoulder, elbow joints, and trunk and back muscle groups, compared with BFRT-F and, in some measurements, BFRT-P. BFRT-P also significantly increased peak torque compared to BFRT-F. BFRT-F demonstrated significant gains in peak torque for multiple joint flexors and extensors. 3) Muscle circumference increased significantly in HIRT and BFRT-P groups, with the highest gain in HIRT. 4) Only HIRT and BFRT-P significantly increased muscle mass, with HIRT demonstrating the highest growth in both arms.

**Conclusion:**

The efficacy hierarchy was HIRT > BFRT-P > BFRT-F. While HIRT is optimal for strength and hypertrophy, BFRT-P is a viable alternative for individuals contraindicated to high-intensity training.

## 1 Introduction

Blood Flow Restriction Training (BFRT), also referred to as KAATSU Training, represents an innovative approach to strength training. This method employs specialized pressure equipment to exert external pressure on the limbs during workouts, effectively blocking venous blood flow while partially restricting arterial blood flow. This unique approach subjects the body to a more intense stimulation under specific loads, with the goal of enhancing muscle strength, size, and endurance (Wei et al., 2019; Loenneke et al., 2025) high-intensity resistance training (HIRT) leads to muscle growth in the upper extremities but at the expense of a greater risk of damaging joints, ligaments, or tendons due to this region being more prone to injury. In contrast, low-intensity resistance training implements a low load with high repetition, which is safer but yields less muscle hypertrophy and greater time consumption (Sinnott et al., 2025). BFRT potentially provides a solution as it achieves the muscle growth and strengthening of high-intensity resistance training while safely performing low-intensity resistance training (Sinnott et al., 2025).

In practical applications, the effects of BFRT on various populations have been extensively researched. For untrained young adults, a study by Yu et al. (2020) found that short-term intensive strength training combined with BFRT not only promotes skeletal muscle growth and improves body composition in adult males but also enhances their resting left ventricular ejection fraction and cardiac output, further bolstering cardiovascular health. In elderly individuals, research by Arnason et al. (2025) revealed the positive impacts of BFRT. They conducted a 6-week BFRT program with elderly participants, resulting in significant increases in quadriceps muscle strength and improved knee flexion and extension abilities. Studies have shown that BFRT can enhance maximum strength and improve body composition in specific muscle groups, providing robust support for athletes’ physical training and competitive performance (LI et al., 2019; Arnason et al., 2025). In the field of rehabilitation, BFRT has also demonstrated its unique value. A study by Kilgas et al. (2019) pointed out that, for patients undergoing anterior cruciate ligament reconstruction, BFRT intervention effectively improves quadriceps function, increases the thickness of the rectus femoris and vastus lateralis muscles, and enhances knee extensor strength, contributing to the patients’ rehabilitation process. Furthermore, BFRT plays an effective role in disease prevention. Study found that BFRT can improve bone health and exercise capacity in elderly individuals without causing exercise-related injuries (Wilson et al., 2013; Han et al., 2024).

Previous studies have demonstrated that blood flow restriction training (BFRT) effectively enhances muscular strength and hypertrophy while reducing mechanical load. Critical implementation parameters include maintaining exercise intensity at 20%–40% 1RM with high-volume protocols (75 total repetitions: 1 × 30 + 3 × 15), administering two to three sessions weekly (Patterson et al., 2019), and applying 50%–80% arterial occlusion pressure (AOP) during the training sessions to optimize training efficacy (Das and Paton, 2022). The scientific community remains divided regarding cuff pressurization methodologies, with proponents for both absolute (Martín-Hernández et al., 2013; Early et al., 2020; Horiuchi et al., 2023) and progressive pressure approaches (Thiebaud et al., 2013; Bemben et al., 2022).

Emerging evidence indicates differential physiological impacts of cuff application techniques (Chang et al., 2023; Chang et al., 2024), suggesting that progressively increasing pressure may induce greater and more sustained metabolic stress (e.g., hypoxia, metabolite accumulation) throughout the intervention period compared to a fixed pressure, potentially enhancing training adaptations (McLeay et al., 2012). Yet controlled comparisons of their effects on upper extremity adaptations remain conspicuously absent in the literature. Based on these knowledge gaps, we hypothesize that: (1) High-load resistance training (HL-RT; 70% 1RM) will demonstrate superior strength and hypertrophic gains compared to both BFRT modalities; (2) Progressive-pressure BFRT (BFRT-P) will surpass fixed-pressure BFRT (BFRT-F) through this enhanced sustained metabolic accumulation. Therefore, the primary objective of this study was to directly compare the effects of HIRT, fixed-pressure BFRT (BFRT-F), and progressive-pressure BFRT (BFRT-P) on enhancing upper limb muscle strength (maximal and isokinetic), muscle circumference, and muscle mass in untrained individuals. We specifically aimed to elucidate whether progressive pressure application offers a mechanistic advantage over fixed pressure within the BFRT paradigm.

## 2 Materials and methods

### 2.1 Participants

This study employed G*Power 3.1 software for sample size estimation. Based on a repeated - measures two - way analysis of variance (ANOVA) design (between - subjects factor: three training modes; within - subjects factor: pre -/post - measurement), a large effect size was set (f = 0.40), referencing the Cohen’s d values (1.19–2.99) for changes in maximal strength from previous experiments conducted by Chang et al. (2024). Other parameters were set as follows: α = 0.05, power = 0.80, number of groups = 3, number of measurements = 2, and the correlation coefficient for repeated measures ρ = 0.5. The calculation results indicated that a total sample size of 24 participants was required. Taking into account potential dropout rates and missing data, 39 participants were randomly recruited. They ranged in age from 18 to 28 and had no prior systematic resistance training experience. Individuals were excluded if they had recently taken medications, had a history of cardiovascular, cerebrovascular, or musculoskeletal diseases, or other exercise contraindications; had a known history of peripheral nerve injury, cardiovascular disease, lung disease, metabolic disease, musculoskeletal injury, or smoking; or could not commit to good attendance, such as being late, leaving early, or skipping sessions. The personal information of all participants was kept confidential. Throughout the research intervention, participants had the right to withdraw from the study at any time for personal reasons. All participants voluntarily signed the “Informed Consent Form” and completed the “Physical Activity Readiness Questionnaire (PAR-Q).” The study adhered to the principles outlined in the Declaration of Helsinki, received ethical approval from the Ethics Committee of Zhejiang Normal University (ZSRT2023079), and was registered with the Chinese Clinical Trial Registry (ChiCTR2300073191).

**FIGURE 1:**
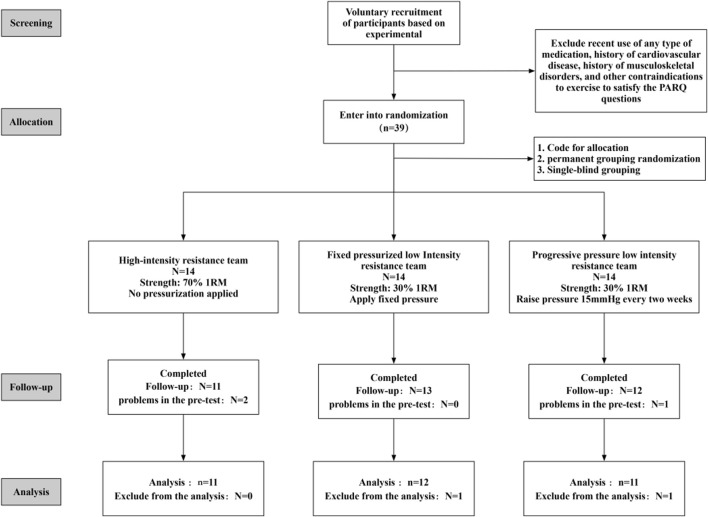
Participant screening flowchart.

Ultimately, 34 valid participants (16 males and 18 females, as illustrated in Figure 1) with an average age of 22.4 years were enrolled in the study. The randomization process involved stratifying participants by gender (male and female) and then randomly assigning them within each gender stratum to the HIRT group, BFRT-F group, and BFRT-P group, ensuring a similar gender ratio across all groups. Baseline characteristics of the participants are presented in Table 1. A one-way ANOVA was conducted to analyze these characteristics, and the results indicated no significant differences between the groups (P > 0.05).

**TABLE 1:** Baseline characteristics of participants.

Variable	HIRT	BFRT-F	BFRT-P	Total	P
Gender (Male/Female)	5/6	6/6	5/6	16/18	P = 0.406
Age (years)	21.81 ±1.53	22.83 ±1.69	22.54 ±1.75	22.41 ±1.67	P = 0.340
Height (cm)	169.81 ±9.57	168.58 ±6.45	167.09 ±7.8 9	168.5 ± 7.86	P = 0.730
Weight (kg)	63.48 ± 9.8	57.48 ±9.02	60.40 ± 9.64	60.18 ± 9.76	P = 0.331
BMI (kg/m^2^)	21.88 ±2.26	20.15 ±2.20	21.53 ±1.91	21.07 ± 2.31	P = 0.137

### 2.2 Training protocols

#### 2.2.1 Pilot study

A 2-week pilot study was conducted to assess and refine key components of the experimental protocol, including cuff pressure levels and training plans, by utilizing the actual training experiences of four non-formal participants (who did not take part in the subsequent formal experiment). Training was scheduled for Monday, Wednesday, and Friday afternoons each week, resulting in a total of three training sessions per week.

#### 2.2.2 Formal experiment

**FIGURE 2:**
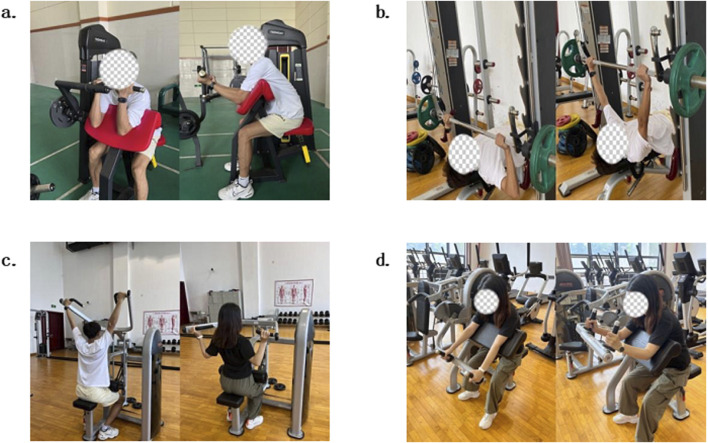
Photos of Training Exercises. **(A)** Bicep Curls, **(B)** Bench Press, **(C)** Lat Pulldowns, **(D)** Tricep Extensions.

Intervention Period: The experimental intervention spanned 8 weeks, with training sessions conducted on Monday, Wednesday, and Friday afternoons each week, lasting 25 min apiece. Each session encompassed a 5-min warm-up, 15 min of main training, and a 5-min cool-down and stretching period. Prior to the commencement of training, participants engaged in a standardized 5-min warm-up until they reached a heart rate of 120–140 bpm. The warm-up protocol consisted of the following phases: 3–4-Minute Light Jog: Participants performed a light jog on a treadmill to gradually elevate heart rate. Dynamic Activation (1 min): Arm Circles (20 s): Forward and backward rotations with extended arms to mobilize shoulder joints. Shoulder Activation (20 s): Banded external rotations (elbows bent at 90°, palms facing upward) to engage rotator cuffs. Thoracic Rotations (20 s total, 10 s per side): Seated or standing with hands clasped behind the head, participants rotated their upper torso laterally to enhance thoracic spine mobility. Under professional supervision, participants then executed the following resistance training exercises in a predetermined sequence: bicep curls, triceps extensions, lat pulldowns, and bench press (as depicted in Figure 2), with the exercise targets outlined in Table 2. In the initial 12 training sessions, the training load was adjusted based on individual responses. The subsequent 12 sessions were modified according to Week four muscle strength test results to optimize effectiveness. All movements were tempo-controlled by a metronome, ensuring concentric and eccentric phases were completed within 2 s to standardize training efficacy.

**TABLE 2:** Primary muscle groups targeted by training exercises.

Training exercises	Primary muscle groups targeted for exercise
Bicep Curls	Biceps brachii
Tricep Extensions	Triceps brachii
Lat Pulldown	Latissimus dorsi
Bench Press	Chest muscles

The training was segmented into three distinct protocols:

HIRT Group: The training load for each exercise was set at 70% of the participant’s 1RM. No external pressure was applied during sessions. Participants performed three sets of eight repetitions per exercise, with 60-s rest intervals between sets.

BFRT-F Group: A lower intensity (30% 1RM) was used for all exercises. A fixed cuff pressure of 115 mmHg and a binding pressure of 30–40 mmHg were maintained throughout training. The protocol comprised four sets: the first set of 30 repetitions followed by three sets of 15 repetitions, with 30-s rests between sets.

BFRT-P Group: similar to BFRT-F, exercises began at 30% 1RM but employed progressive pressure: starting at 90 mmHg in Week one and increasing by 15 mmHg every 2 weeks. The binding pressure (30–40 mmHg) matched the BFRT-F group. Set structure (4 sets: 30 + 15+15 + 15 reps) and rest intervals (30 s) were identical.

Participants in the BFRT groups wore specialized pressure cuffs (KAATSU Master, Sato Sports Plaza, Japan) connected to portable smart BFRT instruments (Yidongkang Intelligent Pressure Training Instrument, ZNJY-01, China). The KAATSU Master cuffs featured a standardized 3-cm width, with an outer nylon fiber layer and an inner medical-grade silicone bladder. Following warm-up, participants assumed a seated position for cuff application. The single-chamber, straight-shaped cuffs were positioned at the proximal third of the upper arm, adjacent to the shoulder joint. During training, the equipment automatically maintained preset pressure levels (determined based on previous research by Qu et al. (2019); Wei et al. (2019)) while allowing real-time adjustments. Pressure was continuously applied throughout each exercise session but immediately released upon completion, with total pressurized training time strictly limited to 15 min per protocol.

### 2.3 Muscular strength testing

Maximum strength (1RM) testing was conducted using resistance training equipment. The test items included arm flexion and extension, arm curls, bench press, and lat pulldown. After a thorough warm-up, subjects gradually increased the weight they lifted until they could no longer lift it, determining the final 1RM value. The warm-up process included running on a treadmill for 5–10 min and several sets of light-weight dynamic stretching exercises to enhance blood circulation in the joints and muscles. Testing began with a weight that the subject could typically lift 5–10 times as a starting point, and then, with adequate rest, the weight was increased until the subject could not complete 10 repetitions, which was considered the submaximal repetition strength. The 1RM value was then estimated using the submaximal repetition weight data and the 1RM calculation method described (Brzycki, 1993). The calculation formula was: 1RM = 100 × submaximal repetition weight/(102.78–2.78 × number of repetitions to failure).

### 2.4 Relative peak torque testing of isokinetic muscle strength

The relative peak torque testing of isokinetic muscle strength was conducted using the isokinetic muscle strength testing and training system (IsoMed 2000; Germany) with standardized protocols. For shoulder testing, participants were positioned supine with the shoulder abducted to 90° ± 2°. The grip width was set to 110% of the acromiohumeral distance. The axis of the dynamometer was aligned with the coracoid process. Gravitational compensation was determined from three preconditioning trials; Elbow testing utilized a seated posture with 110° ± 5° hip flexion, laser-guided radioulnar alignment to maintain neutral forearm rotation, and lever arm length standardized to 85% forearm length (styloid process to olecranon); Trunk testing adopted a seated position with 60° ± 3° hip flexion, rotation axis centered between greater trochanter and ischial tuberosity, and 5 Nm preload torque applied. Angular velocities (60°/s and 180°/s) were randomized via block randomization, with five repetitions at 60°/s and 10 repetitions at 180°/s. Relative peak torque normalized to body weight was recorded throughout trials.

### 2.5 Arm circumference testing

In this study, a tape measure was used to test the arm circumference of both arms. Before testing, subjects were instructed to avoid high-intensity exercise for the past 3 days to reduce the impact of muscle congestion on the test results. The test mainly measured the circumference of both arms in both contracted and relaxed states using a mechanical anthropometric tape measure with a precision of 0.1 cm. To reduce the influence of visuomotor coupling, triplicate measurements were taken and averaged. Reliability was confirmed through intra-rater ICCs >0.98 (95%CI: 0.95–0.99) from triplicate measurements in 15 pilot subjects, with SEM = 0.13 cm.

### 2.6 Muscle mass testing

In this study, the InBody body composition analyzer (TANITA BC-720, Tanita Corporation, Tokyo, Japan) was used to test the muscle mass of both arms. The test mainly measured indicators such as the subjects’ BMI and muscle mass of both arms using multi-frequency bioelectrical impedance analysis (BIA). Muscle mass measurements were conducted in the morning after an overnight fast. To ensure accuracy, participants were instructed to empty their bladders, avoid eating and drinking, and refrain from engaging in strenuous physical activity prior to the test. Before the test, the instrument was thoroughly calibrated. Participants were asked to remove their outer clothing, shoes, and any metal objects on their bodies. They stood at the designated position on the body composition analyzer, with their arms held at approximately a 45 - degree angle from their bodies, looking straight ahead, maintaining a normal upright posture, and remaining motionless until the test was completed. Considering that environmental factors could interfere with the test results, triplicate measurements were taken and averaged to improve the accuracy of the test.

### 2.7 Data analysis methods

After completing the experiments, all raw data collected from this study were entered into Excel 2010 software for storage and organization. Subsequently, the data from Excel were imported into IBM SPSS Statistics 27.0 software for comprehensive statistical analysis. The statistical analysis encompassed the following key aspects:

Baseline descriptive statistics were calculated for individual factors, including participants’ sex, age, height, and weight, and were presented as mean ± standard deviation. One-way ANOVA was employed to assess significant differences among the groups. Descriptive statistics were also computed for the pre-test and post-test values of crucial dependent variables, such as maximum strength (1RM), limb circumference, muscle mass, and the flexor and extensor muscle groups of the upper limb shoulder and elbow joints, as well as the lumbar and dorsal regions. All data were first subjected to a normality test.

A two-way repeated measures ANOVA (Group × Time) was utilized to assess the main effects of group and time, as well as their interaction. Post-hoc pairwise comparisons with Bonferroni correction were conducted when significant interactions or main effects were detected. The magnitude of effect sizes was reported using Cohen’s d. the effect sizes were classified into three categories: small effect size (0.2 ≤ Cohen’s d < 0.5), medium effect size (0.5 ≤ Cohen’s d < 0.8), and large effect size (Cohen’s d ≥ 0.8). Additionally, one-way ANOVA was used to determine significant differences in the magnitude of increase between the groups. For all statistical tests, *P* < 0.05 was considered significant, and *P* < 0.01 was considered highly significant.

## 3 Results

### 3.1 Maximal strength results

Significant Group × Time interactions (F = 4.716–8.946, *P* < 0.05) and main effects of time (F = 47.759–106.463, *P* < 0.01) were observed across all exercises (Table 3). Post-hoc analyses revealed that in the HIRT group, there were significant increases (*P* < 0.05) in one-repetition maximum (1RM) strength for the bicep curl, tricep extension, lat pulldown, and bench press exercises, with the highest increase of up to 56.4% (observed in the tricep extension). In the BFRT-P group, there were significant increases (*P* < 0.01) in 1RM strength for all exercises, with gains ranging from 31.2% to 40.4%, but no significant differences were found compared to the HIRT group (*P* > 0.05). In the BFRT-F group, the lat pulldown (*P* < 0.05), Tricep Extensions (*P* < 0.01) and bench press (*P* < 0.05) showed significant increases, with the smallest gains (ranging from 9.1% to 16.7%), which were significantly lower than those in the HIRT and BFRT-P groups (*P* < 0.05).

**TABLE 3:** Results of Difference Tests for Maximal Strength Indicators Across Groups (Unit: kg).

Variable	Group	Pre-test	Post-test	Interaction	Main effect		Cohen d	95%CI		Change%
					Group	Time		Lower	Upper	
Bicep Curls	HIRT	21.64 ± 5.69	28.29 ± 7.67^**^	4.716^#^	0.680	47.759^#^	1.284	−9.157	−4.135	32.3%^@@^
	BFRT-F	21.78 ± 4.04	23.64 ± 4.69				0.748	−4.268	0.541	9.1%
	BFRT-P	20.71 ± 2.60	26.74 ± 4.37^**^				1.415	−8.535	−3.512	31.2%^@@^
Tricep Extensions	HIRT	21.43 ± 6.08	32.68 ± 6.79^**^	8.946^#^	0.526	106.463^#^	3.221	−13.900	−8.597	56.4%^@@^
	BFRT-F	22.87 ± 5.21	26.55 ± 6.60^**^				0.968	−6.214	−1.137	14.2%
	BFRT-P	20.27 ± 6.67	28.25 ± 10.86^**^				1.469	−10.636	−5.333	40.4%^@@^
Lat Pulldowns	HIRT	53.61 ± 11.15	79.57 ± 17.03^**^	8.024^#^	0.899	86.143^#^	2.444	−32.851	−19.073	49.5%^@@^
	BFRT-F	52.52 ± 15.23	60.18 ± 16.95^*^				0.694	−14.255	−1.064	16.7%
	BFRT-P	52.49 ± 22.74	72.40 ± 28.39^**^				1.669	−26.808	−13.030	18.3%^@@^
Bench Presses	HIRT	38.30 ± 16.43	52.40 ± 20.90^**^	5.988^#^	0.516	91.281^#^	2.006	−18.278	−9.905	38.2%^@@^
	BFRT-F	35.80 ± 18.30	41.33 ± 20.71^*^				0.771	−9.537	−1.521	12.6%
	BFRT-P	39.64 ± 21.06	53.51 ± 26.11^**^				2.257	−18.058	−9.685	35.1%^@@^

^*^Significant difference compared to pre-training (P < 0.05).

^**^Very significant difference compared to pre-training (P < 0.01).

^@^Indicates a significant difference compared to the BFRT-F, group (P < 0.05).

^@@^Indicates a very significant difference compared to the BFRT-F, group (P < 0.01).

^and^Indicates a significant difference compared to the BFRT-P, group (P < 0.05).

^andand^Indicates a very significant difference compared to the BFRT-P, group (P < 0.01). ^#^indicates that the interaction effect or main effect is significant (P < 0.05). The same applies below.

### 3.2 Isokinetic muscle strength results

#### 3.2.1 Relative peak torque results for shoulder flexors and extensors

Significant Group × Time interactions were observed for all shoulder flexor and extensor muscle groups (F = 5.874–21.635, *P* < 0.05), along with significant main effects of time (F = 63.739–231.421, *P* < 0.01) (Table 4). Post-hoc analyses showed that in the HIRT group, there were significant increases (*P* < 0.01) in relative peak torque for all shoulder flexor and extensor muscle groups at angular velocities of 60°/s and 180°/s, with gains ranging from 6.5% to 8.4%. In the BFRT-P group, there were also significant increases (*P* < 0.05) in relative peak torque for all shoulder flexor and extensor muscle groups at these angular velocities, but the magnitude of increase (4.6%–9.1%) was lower than that in the HIRT group (*P* < 0.05). In the BFRT-F group, with the exception of the left arm shoulder joint flexor at 60°/s, there were significant increases (*P* < 0.05) in relative peak torque for all shoulder flexor and extensor muscle groups at 60°/s and 180°/s, with gains ranging from 1.6% to 3.1%, which were significantly lower than those in the HIRT and BFRT-P groups (*P* < 0.05). These findings suggest that, in terms of improving the relative peak torque of shoulder flexors and extensors, the HIRT method is the most effective, followed by the BFRT-P method, while the BFRT-F method is relatively the least effective.

**TABLE 4:** Difference tests for relative peak torque indicators of the shoulder joint (Unit: N·m/kg).

Variable	Group	Pre-test	Post-test	Interaction	Main effect		Cohen d	95%CI		Change%
					Group	Time		Lower	Upper	
Left arm shoulder joint flexor (60°/s)	HIRT	0.43 ± 0.09	0.47 ± 0.08^**^	5.874^#^	0.047	63.739^#^	1.580	−0.042	−0.021	7.6%^@@^
	BFRT-F	0.44 ± 0.10	0.45 ± 0.11				0.647	−0.019	0.000	1.7%
	BFRT-P	0.44 ± 0.07	0.47 ± 0.07^**^				1.829	−0.037	−0.017	6.3%^@@^
Left arm shoulder joint flexor (180°/s)	HIRT	0.32 ± 0.07	0.34 ± 0.08^**^	9.190^#^	0.278	86.926^#^	1.597	−0.035	−0.019	8.3%^@@^
	BFRT-F	0.31 ± 0.09	0.32 ± 0.08				0.725	−0.015	0.000	3.1%
	BFRT-P	0.32 ± 0.07	0.35 ± 0.07^**^				2.512	−0.037	−0.020	9.1%^@@^
Left arm shoulder joint extensor (60°/s)	HIRT	0.67 ± 0.15	0.71 ± 0.15^**^	21.635^#^	0.615	231.421^#^	3.750	−0.048	−0.035	6.5%^@@&^
	BFRT-F	0.67 ± 0.15	0.68 ± 0.14^*^				1.085	−0.019	−0.006	2.0%
	BFRT-P	0.62 ± 0.09	0.64 ± 0.10^**^				3.302	−0.035	−0.022	4.6%^@@^
Left arm shoulder joint extensor (180°/s)	HIRT	0.53 ± 0.13	0.57 ± 0.14^**^	18.885^#^	0.811	141.101^#^	2.641	−0.047	−0.031	7.8%^@@^
	BFRT-F	0.52 ± 0.12	0.52 ± 0.13^*^				0.508	−0.015	0.000	1.2%
	BFRT-P	0.47 ± 0.08	0.5 ± 0.080^**^				5.450	−0.039	−0.023	6.9%^@@^
Right arm shoulder joint flexor (60°/s)	HIRT	0.48 ± 0.07	0.51 ± 0.07^**^	14.988^#^	0.018	139.027^#^	2.611	−0.044	−0.028	7.8%^@@^
	BFRT-F	0.49 ± 0.09	0.5 ± 0.10^*^				0.667	−0.017	−0.002	1.6%
	BFRT-P	0.47 ± 0.06	0.50 ± 0.07^**^				3.183	−0.040	−0.024	6.8%^@@^
Right arm shoulder joint flexor (180°/s)	HIRT	0.36 ± 0.08	0.39 ± 0.09^**^	10.523^#^	0.106	121.121^#^	2.771	−0.039	−0.023	8.4%^@@^
	BFRT-F	0.38 ± 0.08	0.39 ± 0.07^*^				0.741	−0.018	−0.003	3.1%
	BFRT-P	0.37 ± 0.06	0.41 ± 0.06^**^				2.396	−0.041	−0.025	9.1%^@@^
Right arm shoulder joint extensor (60°/s)	HIRT	0.76 ± 0.10	0.81 ± 0.11^**^	7.398^#^	0.050	115.633^#^	3.209	−0.060	−0.037	6.4%^@@^
	BFRT-F	0.79 ± 0.15	0.81 ± 0.17^*^				0.924	−0.030	−0.008	2.1%
	BFRT-P	0.76 ± 0.16	0.80 ± 0.16^**^				1.805	−0.049	−0.025	4.8%^@@^
Right arm shoulder joint extensor (180°/s)	HIRT	0.58 ± 0.10	0.62 ± 0.10^**^	8.511^#^	0.018	133.384^#^	3.109	−0.045	−0.029	6.3%^@@^
	BFRT-F	0.60 ± 0.14	0.62 ± 0.15^**^				0.896	−0.022	−0.006	2.4%
	BFRT-P	0.59 ± 0.08	0.62 ± 0.09^**^				2.412	−0.037	−0.021	4.6%^@@^

#### 3.2.2 Results for relative peak torque of elbow flexors and extensors

Significant Group × Time interactions were observed for all elbow flexor and extensor muscle groups (F = 6.119–25.159, *P* < 0.05), along with significant main effects of time (F = 96.578–259.203, *P* < 0.05) (Table 5). Post-hoc analyses demonstrated that in the HIRT group, there were significant increases in relative peak torque for all elbow flexor and extensor muscle groups (*P* < 0.01), with gains ranging from 6.0% to 8.7%. Notably, the right elbow extensors showed the best performance at 180°/s. In the BFRT-P group, significant increases in relative peak torque were also observed for all elbow flexor and extensor muscle groups (*P* < 0.05), but the magnitude of increase (5.1%–6.8%) was significantly lower than that in the HIRT group (*P* < 0.05). In the BFRT-F group, with the exception of the relative peak torque for the right arm elbow joint extensor at 180°/s, significant increases were observed for all other elbow flexor and extensor muscle groups, with gains ranging from 1.8% to 2.9%, which were significantly lower than those in the other two groups (*P* < 0.05). Once again, it has been shown that in terms of enhancing the relative peak torque of elbow flexors and extensors, the HIRT training method is the most effective, followed by the BFRT-P method, while the BFRT-F training method provides the least benefits.

**TABLE 5:** Difference tests for relative peak torque indicators of elbow muscles (Unit: N·m/kg).

Variable	Group	Pre-test	Post-test	Interaction	Main effect		Cohen d	95%CI		Change%
					Group	Time		Lower	Upper	
Left arm Elbow joint flexor (60°/s)	HIRT	0.45 ± 0.05	0.48 ± 0.05^**^	6.119^#^	0.345	96.578^#^	4.409	−0.039	−0.022	6.8%^@@^
	BFRT-F	0.44 ± 0.06	0.45 ± 0.06^*^				0.639	−0.020	−0.004	2.8%
	BFRT-P	0.44 ± 0.04	0.47 ± 0.03^**^				2.170	−0.038	−0.021	6.8%^@@^
Left arm Elbow joint flexor (180°/s)	HIRT	0.33 ± 0.06	0.35 ± 0.06^**^	6.561^#^	0.438	103.138^#^	5.011	−0.028	−0.016	6.6%^@@^
	BFRT-F	0.32 ± 0.05	0.33 ± 0.05^**^				0.633	−0.014	−0.003	2.9%
	BFRT-P	0.31 ± 0.04	0.34 ± 0.05^**^				2.395	−0.026	−0.014	6.1%^@@^
Left arm Elbow joint extensor (60°/s)	HIRT	0.68 ± 0.13	0.73 ± 0.13^**^	19.453^#^	0.192	235.366^#^	3.771	−0.049	−0.036	6.4%^@@&^
	BFRT-F	0.68 ± 0.06	0.69 ± 0.07^**^				1.113	−0.020	−0.007	2.0%
	BFRT-P	0.68 ± 0.07	0.71 ± 0.07^**^				3.402	−0.038	−0.025	4.6%^@@^
Left arm Elbow joint extensor (180°/s)	HIRT	0.56 ± 0.11	0.59 ± 0.11^**^	10.471^#^	0.744	100.265^#^	3.795	−0.042	−0.024	6.0%^@@^
	BFRT-F	0.53 ± 0.07	0.54 ± 0.06^*^				0.461	−0.017	−0.001	1.8%
	BFRT-P	0.53 ± 0.07	0.56 ± 0.08^**^				2.645	−0.040	−0.022	5.7%^@@^
Right arm Elbow joint flexor (60°/s)	HIRT	0.47 ± 0.07	0.50 ± 0.08^**^	25.159^#^	0.034	259.203^#^	3.677	−0.037	−0.027	6.7%^@@&^
	BFRT-F	0.47 ± 0.06	0.48 ± 0.06^**^				1.080	−0.014	−0.004	2.0%
	BFRT-P	0.47 ± 0.05	0.50 ± 0.06^**^				3.807	−0.030	−0.020	5.1%^@@^
Right arm Elbow joint flexor (180°/s)	HIRT	0.33 ± 0.06	0.36 ± 0.07^**^	10.535^#^	1.721	120.805^#^	2.654	−0.030	−0.019	7.2%^@@^
	BFRT-F	0.37 ± 0.04	0.38 ± 0.05^*^				0.672	−0.013	−0.002	1.9%
	BFRT-P	0.33 ± 0.03	0.35 ± 0.04^**^				3.058	−0.026	−0.015	6.2%^@@^
Right arm Elbow joint extensor (60°/s)	HIRT	0.75 ± 0.03	0.80 ± 0.04^**^	11.766^#^	0.037	136.244^#^	2.768	−0.060	−0.038	6.4%^@@^
	BFRT-F	0.76 ± 0.07	0.78 ± 0.08^*^				0.791	−0.026	−0.005	1.8%
	BFRT-P	0.75 ± 0.05	0.80 ± 0.05^**^				2.692	−0.053	−0.031	5.4%^@@^
Right arm Elbow joint extensor (180°/s)	HIRT	0.55 ± 0.07	0.60 ± 0.08^**^	11.944^#^	0.273	100.644^#^	2.803	−0.060	−0.037	8.7%^@@&^
	BFRT-F	0.58 ± 0.09	0.59 ± 0.09				0.628	−0.022	−0.001	2.2%
	BFRT-P	0.54 ± 0.05	0.58 ± 0.07^**^				1.827	−0.045	−0.022	5.9%^@@^

#### 3.2.3 Relative peak torque results for lumbar flexors and extensors

Except for the peak torque of the Lumbar extensors at 60°/s, significant Group × Time interactions were observed for the peak torque of lumbar extensor muscle groups (F = 4.223–10.728, *P* < 0.05). The main effect of time was also significant, with significant increases in peak torque observed for all lumbar extensor muscle groups (F = 6.912–22.889, *P* < 0.05) (Table 6). Post-hoc analyses demonstrated that in the HIRT group, there were significant increases in peak torque for both lumbar flexor and extensor muscle groups at 60°/s and 180°/s (*P* < 0.01), with gains ranging from 2.6% to 3.1%. In contrast, the BFRT-F and BFRT-P groups showed no significant changes in lumbar muscle group peak torque (*P* > 0.05). The HIRT group had a significantly higher magnitude of increase compared to the BFRT-F and BFRT-P groups in tests of peak torque for lumbar flexors at 60°/s and for both lumbar flexors and extensors at 180°/s (*P* < 0.05).

**TABLE 6:** Difference tests for lumbar relative peak torque indicators (Unit: N·m/kg).

Variable	Group	Pre-test	Post-test	Interaction	Main effect		Cohen d	95%CI		Change%
					Group	Time		Lower	Upper	
Lumbar flexors (60°/s)	HIRT	2.51 ± 0.33	2.59 ± 0.32^**^	5.269^#^	0.122	14.886^#^	2.621	−0.110	−0.045	3.1%^@@&&^
	BFRT-F	2.53 ± 0.24	2.54 ± 0.25				0.235	−0.045	0.017	0.6%
	BFRT-P	2.48 ± 0.34	2.50 ± 0.37				0.226	−0.047	0.019	0.5%
Lumbar flexors (180°/s)	HIRT	1.85 ± 0.11	1.91 ± 0.11^**^	4.223^#^	0.042	10.053^#^	1.693	−0.089	−0.030	3.1%^@@&&^
	BFRT-F	1.89 ± 0.26	1.90 ± 0.26				0.165	−0.037	0.019	0.6%
	BFRT-P	1.87 ± 0.16	1.88 ± 0.18				0.180	−0.038	0.020	0.3%
Lumbar extensors (60°/s)	HIRT	3.69 ± 0.21	3.79 ± 0.20^**^	3.241	1.510	6.912^#^	3.140	−0.156	−0.042	2.6%^@@&&^
	BFRT-F	3.80 ± 0.64	3.81 ± 0.67				0.126	−0.069	0.040	0.3%
	BFRT-P	4.06 ± 0.42	4.07 ± 0.43				0.109	−0.069	0.045	0.2%
Lumbar extensors (180°/s)	HIRT	2.89 ± 0.11	3.00 ± 0.12^**^	10.728^#^	0.284	22.889^#^	4.084	−0.141	−0.074	2.6%^@@&&^
	BFRT-F	2.88 ± 0.17	2.90 ± 0.16				0.232	−0.048	0.017	0.3%
	BFRT-P	2.87 ± 0.36	2.88 ± 0.39				0.204	−0.046	0.021	0.2%

### 3.3 Arm circumference results for both arms

A significant main effect of group was observed for the measurements of arm relaxed circumference and left arm contracted circumference (F = 3.422–4.201, *P* < 0.05). There were also significant Group × Time interactions for all arm circumference indicators (F = 4.747–8.854, *P* < 0.05), along with significant main effects of time (F = 38.960–63.361, *P* < 0.01) (Table 7). Post-hoc analyses demonstrated that in the HIRT group, there were significant increases in arm circumference for both the left and right arms in both relaxed and contracted states (*P* < 0.01), and these increases were significantly higher than those in the BFRT-F group (*P* < 0.05), with gains ranging from 6.4% to 8.7%. The changes were more pronounced in the contracted state. In the BFRT-P group, significant increases in arm circumference were observed for both the left and right arms in both relaxed and contracted states (*P* < 0.05), with gains ranging from 3.7% to 6.1%. In the BFRT-F group, there were no significant increases in arm circumference for either the left or right arms in both relaxed and contracted states (*P* > 0.05), with gains ranging from 1.8% to 2.5%, which were significantly lower than those in the HIRT group (*P* < 0.05). These results indicate that the HIRT group exhibited more significant effects in increasing muscle circumference compared to the BFRT-F and BFRT-P groups.

**TABLE 7:** Difference Test Results for Muscle Circumference Indicators (Unit: cm).

Variable	Group	Pre-test	Post-test	Interaction	Main effect		Cohen d	95%CI		Change%
					Group	Time		Lower	Upper	
Left arm relaxed circumference	HIRT	27.88 ± 3.25	29.66 ± 3.12^**@^	6.936^#^	4.201^#^	56.485^#^	3.245	−2.306	−1.253	6.4%^@@^
	BFRT-F	25.05 ± 3.00	25.50 ± 2.81				0.732	−0.952	0.055	1.8%
	BFRT-P	27.48 ± 2.81	28.57 ± 3.52^*^				0.869	−1.611	−0.559	3.9%
Left arm contracted circumference	HIRT	29.94 ± 3.89	32.59 ± 4.53^**@^	6.918^#^	3.643^#^	42.031^#^	2.745	−3.493	−1.824	8.7%^@@&^
	BFRT-F	26.84 ± 3.29	27.42 ± 3.23				0.534	−1.375	0.222	2.5%
	BFRT-P	29.07 ± 3.07	30.36 ± 4.51^*^				0.693	−2.128	−0.459	6.1%
Right arm relaxed circumference	HIRT	27.68 ± 3.16	29.41 ± 3.33^**@^	4.747^#^	3.422^#^	38.960^#^	1.496	−2.359	−1.102	6.4%^@@^
	BFRT-F	25.34 ± 2.86	25.76 ± 2.81				0.533	−1.022	0.182	1.8%
	BFRT-P	26.92 ± 2.19	28.06 ± 2.48^**^				1.029	−1.762	−0.506	4.1%
Right arm contracted circumference	HIRT	29.71 ± 4.07	31.87 ± 4.48^**@^	8.854^#^	3.058	63.361^#^	2.838	−2.746	−1.587	7.2%^@@&^
	BFRT-F	26.94 ± 2.97	27.46 ± 3.15				0.609	−1.072	0.038	1.9%
	BFRT-P	28.22 ± 3.21	29.40 ± 3.01^**^				1.004	−1.759	−0.600	3.7%

### 3.4 Muscle mass results for both arms

A significant Group × Time interaction was observed for muscle mass (F = 6.414–9.010, *P* < 0.05), along with a significant main effect of time (F = 20.551–35.301, *P* < 0.05) (Table 8). Post-hoc analyses demonstrated that in the HIRT group, there were significant increases in muscle mass for both the left and right arms (*P* < 0.01), with a 15.1% increase in the left arm and a 22.6% increase in the right arm. In the BFRT-P group, there were also significant increases in muscle mass for both the left and right arms (*P* < 0.05), with a 5.2% increase in the left arm, which was significantly lower than that in the HIRT group (*P* < 0.05), and a 10.6% increase in the right arm. In the BFRT-F group, there were no significant changes in muscle mass (*P* > 0.05), but the increases (2.5%–2.9%) were significantly lower than those in the HIRT group (*P* < 0.01).

**TABLE 8:** Difference Tests for Muscle Mass Indicators (Unit: kg).

Variable	Group	Pre-test	Post-test	Intera ction	Main effect		Cohen d	95%CI		Change%
					Group	Time		Lower	Upper	
Left arm muscle mass	HIRT	2.48 ± 0.70	2.83 ± 0.70^**^	9.010^#^	0.677	35.301^#^	1.453	−0.447	−0.240	15.1%^@@&^
	BFRT-F	2.35 ± 0.50	2.41 ± 0.52				0.610	−0.162	0.037	2.9%
	BFRT-P	2.37 ± 0.63	2.48 ± 0.60^*^				0.764	−0.213	−0.006	5.2%
Right arm muscle mass	HIRT	3.32 ± 1.28	3.98 ± 1.28^**^	6.414^#^	0.859	20.551^#^	1.365	−0.928	−0.399	22.6%^@@^
	BFRT-F	3.01 ± 1.17	3.03 ± 1.10				0.058	−0.274	0.232	2.5%
	BFRT-P	3.00 ± 1.13	3.32 ± 1.33^*^				0.724	−0.584	−0.055	10.6%

## 4 Discussion

To our knowledge, this study represents the first controlled comparison of BFRT-P, BFRT-F, and HIRT on upper limb muscle strength and hypertrophy. Our findings directly align with the two primary hypotheses proposed. First, as hypothesized (Hypothesis 1), HIRT demonstrated unequivocal superiority in enhancing maximal strength and hypertrophy. The HIRT group exhibited the most pronounced gains across all exercises, including a 56.4% increase in tricep extension 1RM and a 22.6% elevation in right arm muscle mass. These results reinforce HIRT’s role as the gold standard for mechanical tension-driven adaptations (Schoenfeld et al., 2017), consistent with our prediction that high-load training would outperform both BFRT modalities. Second, supporting Hypothesis 2, BFRT-P surpassed BFRT-F in efficacy. Despite identical exercise loads (30% 1RM), the BFRT-P group achieved markedly greater strength improvements (31.2%–40.4%↑ vs 9.1%–16.7%↑ in BFRT-F) and morphological adaptations (e.g., 10.6%↑ right arm muscle mass vs non-significant 2.9%↑ in BFRT-F). This divergence likely stems from BFRT-P’s progressive pressure protocol, which amplifies metabolic stress through cumulative hypoxia and metabolite retention (McLeay et al., 2012), aligning with our hypothesis that sustained metabolic accumulation underlies BFRT-P’s advantages.

Isokinetic assessments further validated this hierarchy: HIRT induced the largest increases in relative peak torque for shoulder (6.5%–8.4%↑) and elbow (6.0%–8.7%↑) muscle groups, while BFRT-P outperformed BFRT-F (e.g., 9.1%↑ vs 3.1%↑ in shoulder flexor torque at 180°/s). Morphologically, HIRT-driven gains in arm circumference (6.4%–8.7%↑) and muscle mass dwarfed those of BFRT-P (3.7%–6.1%↑; 5.2%–10.6%↑), with BFRT-F showing minimal improvements (1.8%–2.5%↑; non-significant 2.5%–2.9%↑). These results collectively confirm our hypothesized efficacy gradient: HIRT > BFRT-P > BFRT-F.

### 4.1 Comparative analysis of maximal strength differences

The results of this study reveal that participants in the HIRT group exhibited substantial strength gains across various power training exercises, encompassing bicep curls, tricep extensions, lat pulldowns, and bench presses. These findings corroborate earlier research (Schoenfeld et al., 2015; Androulakis-Korakakis et al., 2021), reinforcing the superiority of HIRT in boosting maximal strength. Moreover, the HIRT group demonstrated notable growth advantages in relative peak torque tests for flexors and extensors of both the left and right shoulder and elbow joints, at angular velocities of both 60°/s and 180°/s, with growth rates surpassing those of the BFRT-F and BFRT-P groups. Additionally, the HIRT group also showcased significantly greater improvements in relative peak torque for flexors and extensors of the lumbar back, compared to the other two groups. These discoveries highlight the benefits of HIRT in enhancing muscle strength, aligning with the research findings of (Lixandrão et al., 2018), which indicate that HIRT yields superior muscle strength gains compared to BFRT. However, studies involving professional athletes suggest that BFRT can achieve comparable muscle strength training effects to HIRT (LI et al., 2019; Wang et al., 2019). This discrepancy may stem from differences in training status among participants, as Geng et al. (2024) discovered that trained individuals are more likely to reap the benefits of BFRT.

In this study, the BFRT-F group utilized a lower load (30% 1RM) and a fixed cuff pressure (115 mmHg) yet still attained significant strength gains in multiple power training exercises, indicating that low-load blood flow restriction training can also contribute to strength improvement. Nonetheless, the magnitude of improvement in the BFRT-F group was less pronounced compared to the HIRT group. Furthermore, prior studies have shown that, from a muscle strength perspective, BFRT with lower cuff pressure has limited benefits in promoting upper limb maximal strength compared to HIRT (Lixandrão et al., 2015). This aligns with our findings, suggesting that BFRT training with lower cuff pressure offers limited advantages in enhancing upper limb maximal strength. Conversely, the BFRT-P group adopted a training approach where cuff pressure was incrementally increased by 15 mmHg every 2 weeks, ultimately reaching 135 mmHg. Experimental results indicate that this group experienced better effects in augmenting maximal strength compared to the BFRT-F group. This may be attributed to the continuous stimulation provided by the progressive cuff pressure adjustment (McLeay et al., 2012), thereby enhancing the training effect to a certain extent. However, the benefits observed in the BFRT-P group were still inferior to those of the HIRT group. This disparity may be related to the age of the participants, as studies have shown that BFRT training protocols are nearly as effective as HIRT protocols in increasing muscle strength among older men (Karabulut et al., 2010). Conversely, Chang et al. (2023) found that older adults are more likely to benefit from HIRT. In conclusion, HIRT demonstrates considerable advantages in enhancing muscle strength, while BFRT-P emerges as a viable alternative, particularly for individuals unable to engage in HIRT. Pressure training can significantly elevate peak torque at specific angular velocities (Chen et al., 2022), and blood flow restriction training can increase heart rate under hypoxic conditions, further enhancing metabolic stress adaptations (Sundblad et al., 2018).

### 4.2 Comparative analysis of muscle morphology differences

The findings of this study reveal that, following an 8-week intervention training program, the HIRT group experienced notable increases in both arm muscle circumference and muscle mass. This underscores the potential benefits of high-intensity training in enhancing muscle physiological attributes. The substantial gains in arm muscle circumference and mass observed in the HIRT group are consistent with the research conducted by Aagaard et al. (2001), which demonstrated that HIRT can stimulate muscle hypertrophy and augment muscle cross-sectional area. However, it is noteworthy that hypertrophy is achievable across a wide load spectrum when training to failure, as emphasized by recent meta-analyses (Currier et al., 2023). BFRT-Induced Hypertrophy Mechanisms: The moderate hypertrophy in BFRT-P (5.2%–10.6% muscle mass gains) likely stems from the proposed enhanced metabolic stress-mediated pathways, including mTOR activation and myogenic satellite cell proliferation (Wei et al., 2019), potentially amplified by the progressively increasing occlusion. In contrast, BFRT-F’s minimal effects (2.5%–2.9%) may reflect insufficient occlusion intensity and potentially suboptimal metabolic stress accumulation over time, as fixed 115 mmHg pressure may not reach the arterial occlusion pressure threshold required for optimal anabolic signaling and this static pressure might induce less cumulative metabolic perturbation than a progressive protocol. Moreover, this study also establishes that the BFRT-P group, to a certain extent, outperformed the BFRT-F group in terms of muscle circumference and mass enhancements, albeit there remains a disparity when compared to the HIRT group. Specifically, the BFRT-P group exhibited statistically significant increases in left arm muscle mass, whereas the BFRT-F group’s muscle mass, though elevated, did not reach statistical significance.

Research by Rolnick et al. (2020) suggests that BFRT can elevate energy expenditure and induce cellular swelling. Kim et al. (2016) found that BFRT significantly augmented the cross-sectional area of distal muscles in both the upper and lower extremities. Conversely, studies by Hansen et al. (2020) and Yasuda et al. (2011) reported that low-intensity blood flow restriction resistance training did not elicit greater muscle fiber growth compared to high-intensity resistance training. Additionally, another study failed to detect a significant difference in muscle cross-sectional area growth between low-intensity blood flow restriction resistance training and conventional high-load training (Farup et al., 2015). In contrast, the results of this study hint that HIRT might yield more pronounced muscle mass gains. These discrepant findings may stem from a multitude of factors, including the specific training program design, participant demographics, varying levels of applied pressure, and diverse muscle circumference and mass measurement techniques.

The *post hoc* power analysis further contextualizes the study’s findings. While the sample size per group was limited, the observed large effect sizes in HIRT-driven adaptations (e.g., 1RM and muscle mass) ensured robust detection of differences. However, the reduced power for medium effects (e.g., BFRT-P vs BFRT-F comparisons) highlights the need for larger-scale trials to validate subtle yet potentially meaningful differences between BFRT protocols.

### 4.3 Limitations of the study

This study has several main limitations. First, the use of fixed absolute pressures (115–135 mmHg) instead of individualized arterial occlusion pressure measurements may attenuate the potential benefits of BFRT-P. Second, although the Brzycki equation provided a pragmatic estimation of 1RM for untrained participants, this indirect measurement introduces greater error compared with direct 1RM testing. Third, the absence of a non-BFRT low-intensity resistance training control group (LIRT) limits our ability to definitively isolate the specific contribution of blood flow restriction itself from the effects of low-load training alone. Future studies should incorporate arterial occlusion pressure calibration, implement direct 1RM assessments, and include appropriate control conditions (e.g., LIRT without BFR) to strengthen ecological validity and isolate the BFR effect.

Despite these limitations, our findings have practical implications. For individuals unable to perform HIRT (e.g., post-surgical patients), BFRT-P offers a viable alternative. Coaches and clinicians should prioritize load intensity (HIRT) and pressure individualization (BFRT-P) to maximize adaptations.

## 5 Conclusion

HIRT exhibits notable superiority in bolstering upper limb strength and enhancing muscle attributes. Conversely, BFRT-P can achieve comparable training outcomes to HIRT under specific circumstances. However, the efficacy of BFRT-F is relatively constrained. Consequently, when crafting training regimens targeted at upper limb strength and muscle hypertrophy, HIRT is typically the more potent option. Nonetheless, considering the unique attributes of BFRT, BFRT-P can serve as a feasible substitute for individuals who are unable to engage in HIRT or for whom HIRT is medically inadvisable.

## Data Availability

The original contributions presented in the study are included in the article/supplementary material, further inquiries can be directed to the corresponding author.
